# Characterization of the amyloid bacterial inclusion bodies of the HET-s fungal prion

**DOI:** 10.1186/1475-2859-8-56

**Published:** 2009-10-28

**Authors:** Raimon Sabaté, Alba Espargaró, Sven J Saupe, Salvador Ventura

**Affiliations:** 1 Departament de Bioquímica I Biologia Molecular and Institut de Biotecnologia i de Biomedicina, Universitat Autònoma de Barcelona, 08193 Bellaterra, Barcelona, Spain; 2 Laboratoire de Génétique Moléculaire des Champignons, IBGC, UMR5095, Université Victor Segalen Bordeaux 2 et CNRS, 1 rue Camille Saint-Saëns, 33077 Bordeaux Cedex, France

## Abstract

The formation of amyloid aggregates is related to the onset of a number of human diseases. Recent studies provide compelling evidence for the existence of related fibrillar structures in bacterial inclusion bodies (IBs). Bacteria might thus provide a biologically relevant and tuneable system to study amyloid aggregation and how to interfere with it. Particularly suited for such studies are protein models for which structural information is available in both IBs and amyloid states. The only high-resolution structure of an infectious amyloid state reported to date is that of the HET-s prion forming domain (PFD). Importantly, recent solid-state NMR data indicates that the structure of HET-s PFD in IBs closely resembles that of the infectious fibrils. Here we present an exhaustive conformational characterization of HET-s IBs in order to establish the aggregation of this prion in bacteria as a consistent cellular model in which the effect of autologous or heterologous protein quality machineries and/or anti-aggregational and anti-prionic drugs can be further studied.

## Background

Protein misfolding and aggregation have become very active areas of research during the last decade. The large efforts devoted in this period to understand the determinants of polypeptide aggregation are justified by the tight connection between the formation of insoluble protein deposits in human tissues and the development of dozens of conformational diseases. These protein deposits are constituted mainly by fibrillar structures known as amyloids with a common cross-β supramolecular organisation [1]. Protein aggregation is also an important problem in biotechnology because during recombinant expression in prokaryotic systems many heterologous proteins misfold and accumulate as insoluble protein deposits named inclusion bodies (IBs) limiting in this way the use of bacteria for the production of therapeutically relevant proteins [2].

IBs formation has long been regarded as an unspecific process relaying on the establishment of hydrophobic contacts [3]. However, an increasing body of evidence suggests that bacterial IBs share a number of common features with the highly ordered and pathogenic amyloid fibrils linked to human diseases [4]. Both processes have been shown to be nucleation driven, sequence specific and lead to the formation of β-sheet enriched structures. However, the detailed structural characterisation of bacterial aggregates is extremely challenging and to which extend a polypeptide embedded in IBs and the same molecule polymerized into amyloids are structurally related has remained essentially unknown.

**Figure 1:**
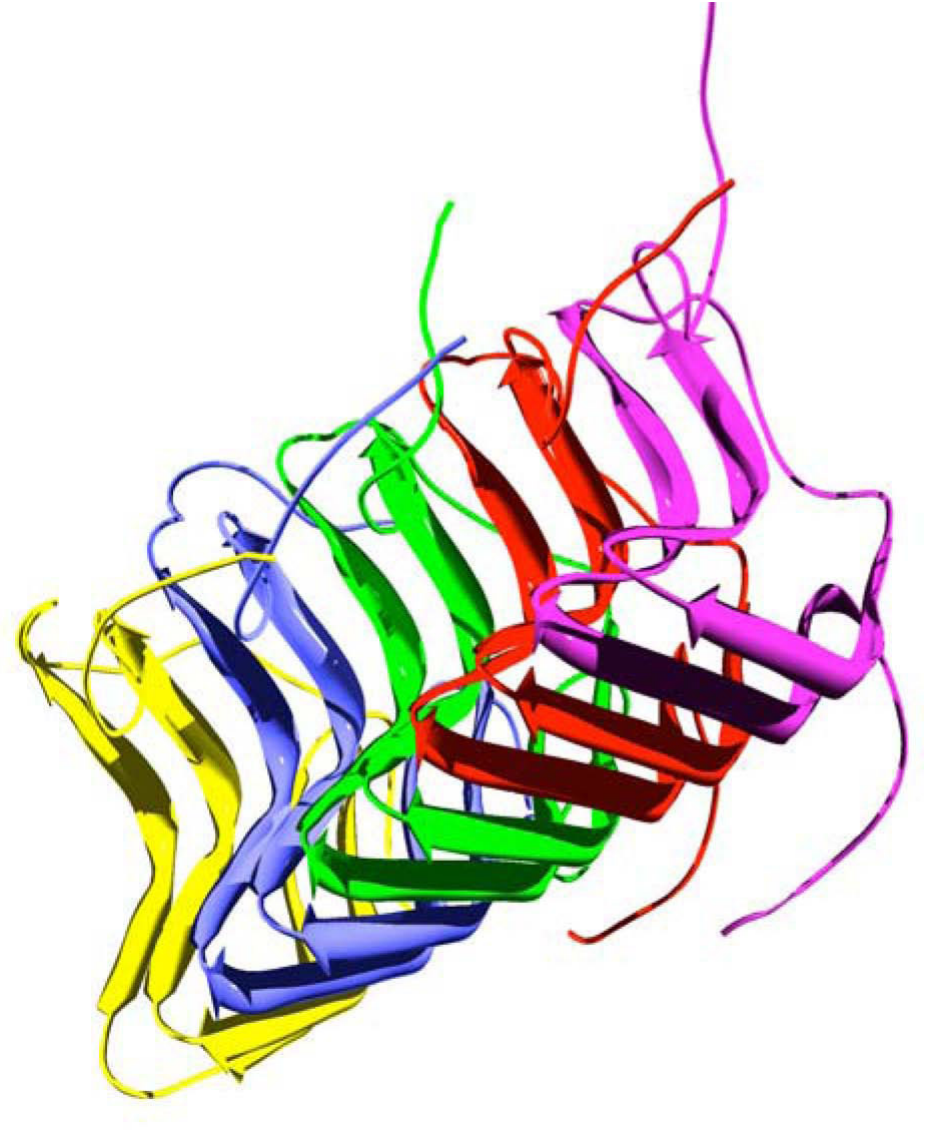
**Three-dimensional structure of the infectious HET-s PFD fibrils**. Ribbon representation the five central molecules corresponding to the lowest energy structure of the HET-s PFD heptamer as calculated from NMR restraints (PDB ID: 2RNM).

Prions represent a particular subclass of amyloids for which the aggregation process becomes self-perpetuating *in vivo *and thus infectious [5]. Fungal prions are infectious filamentous polymers of proteins. Among these prions are the [*PSI*^+^], [URE3] and [*PIN*^+^] yeast prions [6,7] and HET-s that is a prion of the filamentous fungus *Podospora anserina *involved in a fungal specific non-self discrimination phenomenon [8]. HET-s is a 289 residues polypeptide. Residues 1-227 form a well-folded globular domain in the soluble HET-s conformation. In contrast the C-terminal region is highly flexible and unstructured. Previous studies have identified the C-terminal region of HET-s spanning residues 218 to 289 as the prion forming domain (PFD) responsible for amyloid formation and prion propagation [9,10]. *In vivo*, this PFD forms dot like aggregates, whereas a longer version in which the globular domain has been truncated, comprising residues 157-289, forms elongated fibrillar aggregates, suggesting that the ability to adopt this supramolecular organization is conferred *in vivo *by the sequences appended to the amyloid core PFD region [11]. The structure of the infectious amyloid fold of HET-s PFD have been solved recently by solid state NMR and represents the only atomic-resolution structure of an infectious fibrillar conformation reported to date [12]. Under close to physiological conditions, the protein adopts a β-solenoid structure with two layers of β-strand per monomer and a characteristic triangular hydrophobic core (Figure 1) [12]. These *in vitro *assembled fibrils are infectious [13,14]. This prion character is strictly associated to the fibril structure obtained at neutral pH since the highly ordered, but conformationally different, HET-s PFD fibrils formed at acidic pH are not infectious [14,15]. HET-s PFD accumulates as IBs when it is over-expressed in *E. coli*. Recently, Wasmer and co-workers, used H/D exchange and solid-state NMR to characterize the HET-s PFD conformation in IBs, demonstrating that it very closely resembles to that in the fibrils, explaining why HET-s PFD IBs are infectious [16]. These results strongly suggest that bacterial cells expressing the infectious form of this eukaryotic prion could provide a simple and powerful system to study how the autologous or heterologous protein quality machinery modulates the *in vivo *assembly, toxicity and infectivity of amyloids. In addition, this cellular model could be a convenient platform for the screening of generic anti-amyloid or specific anti-prion compounds. Towards these aims we present here an exhaustive biophysical and physicochemical characterization of the IBs formed by the different amyloidogenic forms of HET-s in order to establish which are the conformational signatures of these aggregates in a standard cellular background and culture conditions.

## Results and Discussion

### HET-s PFD assembles into a β-sheet enriched structure in bacterial IBs

**Figure 2:**
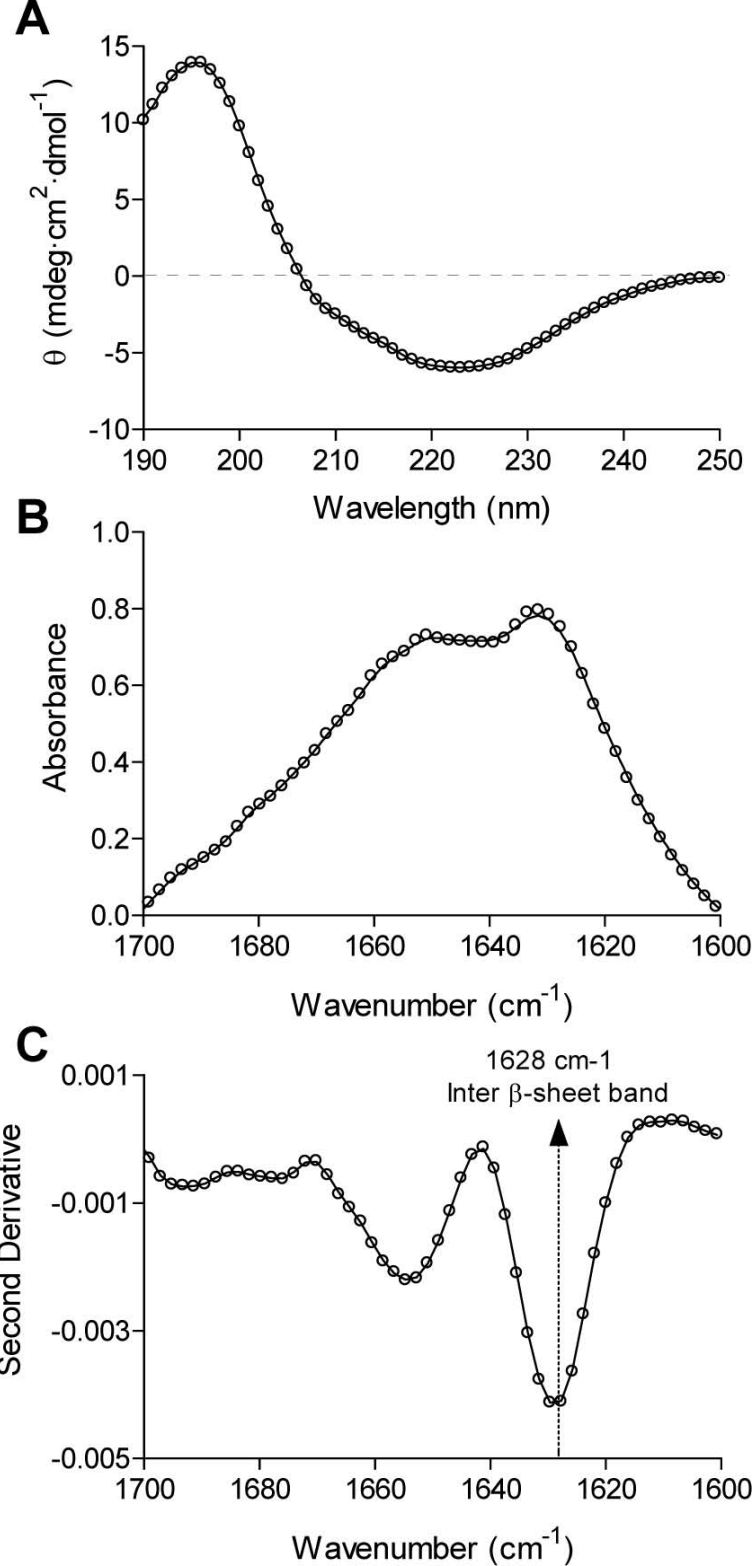
**Secondary structure of HET-s PFD IBs**. (A) Circular dichroism spectra, and (B and C) FTIR absorbance and second derivative spectra in the amide I region of the spectra showing the characteristic spectral bands of β-sheet conformations.

The aggregation of soluble proteins into amyloid fibrils invariably results in an increase in their β-sheet content [1]. In this way, the soluble and unstructured HET-s PFD undergoes a transition towards a β-sheet enriched conformation upon *in vitro *fibrillation [14,17]. The far-UV CD spectrum of HET-s mature fibrils displays a negative band at ~217 nm characteristic of β-sheet structures [14,17]. In the CD spectrum of HET-s PFD IBs the band is shifted 6 nm and centred at 223 nm (Figure 2A). This shift in the β-sheet signal in the far-UV CD spectra of aggregated amyloid proteins has been shown to be related to differences in the macroscopic morphology of the fibrils and thought to arise from the superposition of the aromatic CD band on the classical β-sheet CD spectrum as a result of changes in the stacking of the polypeptide aromatic side-chains in the fibrils [18]. FTIR spectroscopy allows more accurate assignment of the secondary structure elements in protein aggregates than CD. The FTIR spectrum of the infectious fibrillar form of HET-s PFD is dominated in the amide I region by a peak at ~1629 cm^-1^. This signal was associated to the presence of a cross-β-sheet structure in the fibrils [14]. The spectrum of HET-s PFD IBs closely resembles that of the fibrils (Figure 2B and 2C), with a main band at ~1628 cm^-1 ^confirming thus the predominance of a β-sheet architecture in these intracellular aggregates.

### Amyloid properties of HET-s IBs

**Figure 3:**
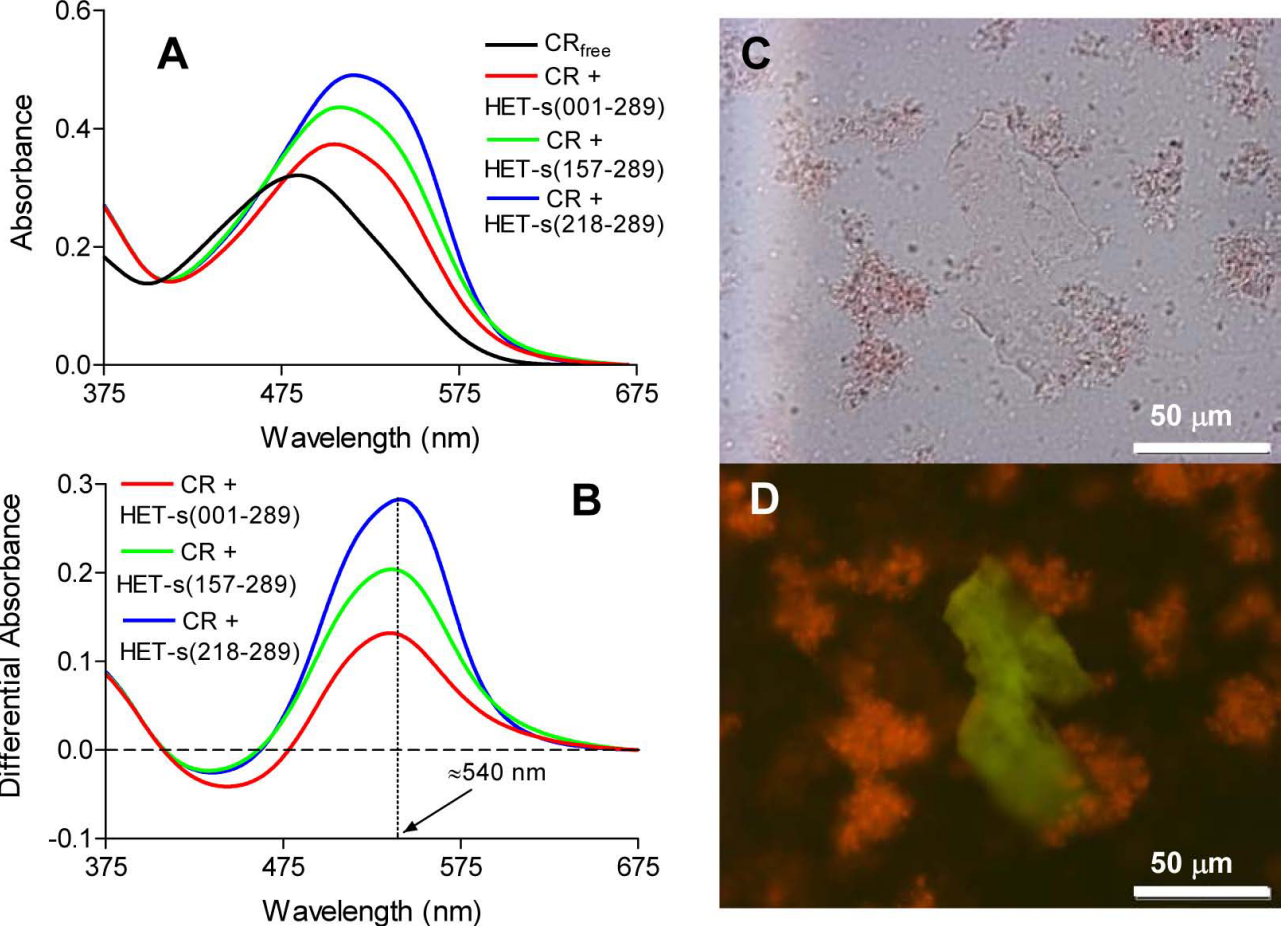
**Congo-Red (CR) binding to different HET-s IBs by UV/Vis spectroscopy and staining and birefringence under cross-polarized light using an optic microscope**. (A and B) CR spectral changes in the presence of different HET-s IBs; (A) Changes in λ_max _and intensity in CR spectra in the presence of IBs; (B) Difference absorbance spectra of CR in the presence and absence of IBs showing in all cases the characteristic amyloid band at ~540 nm. (C) HET-PFD IBs stained with Congo red and observed at 40× magnification and in (D) the same field observed between crossed polarizers displaying the green birefringence characteristic of amyloid structures.

We used the amyloid specific dye Congo Red (CR) to verify that the detected IBs β-sheet structures display typical amyloid properties. The absorbance of CR increases and the spectrum maximum red-shifts to ~508 nm in the presence of HET-s PFD IBs (Figure 3A). This spectral change is identical to that observed in the presence of the infectious fibrils formed by the prion domain [14]. In addition, the difference spectrum between the dye in the presence and absence of IBs allows detecting the characteristic amyloid band at ~540 nm (Figure 3B). HET-s amyloid fibrils [19] exhibit CR birefringence, which is accepted to be one of the most stringent diagnostics for amyloid conformation [1]. As shown in Figure 3C and 3D, HETs PFD IBs also show a strong green-gold birefringence upon incubation with CR and illumination under cross-polarized light.

The structural properties of the bacterial IBs formed by the full-length HET-s prion and HET-s (157-289) have not been explored previously. However, it was shown that they display a common proteinase K (PK) resistant core which likely corresponds to the PFD region [16]. Therefore, one might expect that the IBs formed by these two proteins would also display amyloid features. In agreement with this hypothesis, both types of IBs bind CR (Figure 3A and 3B). Interestingly, the change in the CR signal correlates with the proportion of amyloidogenic versus non-amyloidogenic regions in the polypeptides. In this way, the CR signal in the presence of PFD IBs is three fold that of the dye in the presence of the full-length prion IBs. The IB-stretch hypothesis postulates that not necessarily all the polypeptide chain is involved in the network of contacts that sustain the β-core of an IB but rather that specific contacts between certain aggregation-prone regions keep the aggregate in a compact state [20]. For HET-s, the data suggest that the PFD is responsible for maintaining the detected IBs β-sheet architecture, as likely happens in the aggregates formed by the prion in its physiological environment.

A striking characteristic of HET-s PFD infectious fibrils is that, in contrast to most amyloid structures, they do not induce Thioflavin-T (Th-T) fluorescence [14], a dye widely used for amyloid diagnostic. Interestingly, in contrast with the IBs formed by other amyloidogenic peptides like the Aβ peptide [21], none of the three assayed HET-s IBs specifically induces Th-T fluorescence (data not shown). Overall, HET-s IBs display affinities for amyloid dyes that closely resemble that of the protein in its infectious fibrillar form.

### The β fibrillar core of HET-s IBs

**Figure 4:**
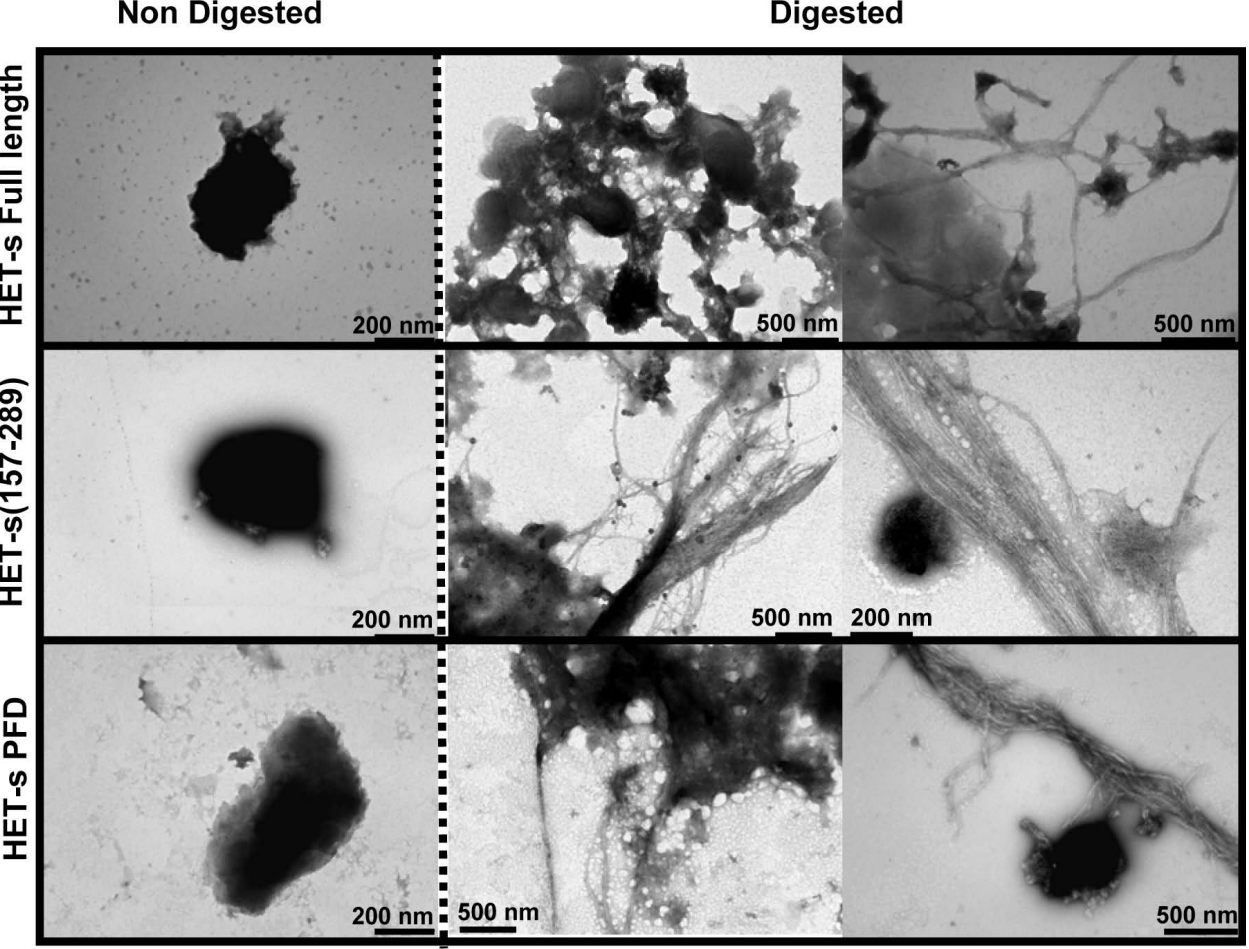
**HET-s IBs structure before (left panel) and after 30 min of proteinase K digestion (central and right panel) as monitored by transmission electronic microscopy**.

Proteinase K is a protease usually used to map the protected core of amyloid fibrils because in spite of being highly active against peptidic bonds it cannot attack the highly packed backbones in amyloid β-sheet structures. We have shown that PK digestion also allows to reveal the existence of a fibrillar core in Aβ peptide IBs [21]. We used the same approach to asses if the presence of a similar fibrillar material might account for the amyloid conformational properties of HET-s IBs. All purified HET-s IBs displayed a typical compact and electrodense structure (Figure 4, left panel). The progress of the digestion reaction was followed by monitoring the changes in the solution turbidity at 350 nm. The reaction reached a plateau after ~60 min (data not shown). We imaged samples taken at *t*_1/2_. The aggregates were partially digested and the presence of abundant fibrillar structures could be observed in the IBs formed by all HET-s polypeptides (Figure 4, central and right panel). It is important to note that amyloid fibrils do not form spontaneously from soluble HET-s in the presence of the PK concentrations used to digest IBs [see Additional file 1] and therefore that the observed fibrillar material is not the result of the release HET-s fragments into solution and its subsequent reassembly. The fibrils are associated with apparently amorphous material and in some micrographs fibrils emerging from the preformed compact IBs are seen. The fibrils dimensions and morphology are very similar to that of the infectious amyloid fibrils formed *in vitro *at pH 7.0: the elementary fibrils are ~5 nm in diameter and tend to associate laterally into bundles or stacks [14,22]. Overall, it appears that the different HET-s IBs constitute a bacterial reservoir of amyloid structures that coexist with more disordered and PK susceptible protein regions.

### Stability of HET-s PFD IBs towards chemical denaturation

**Figure 5:**
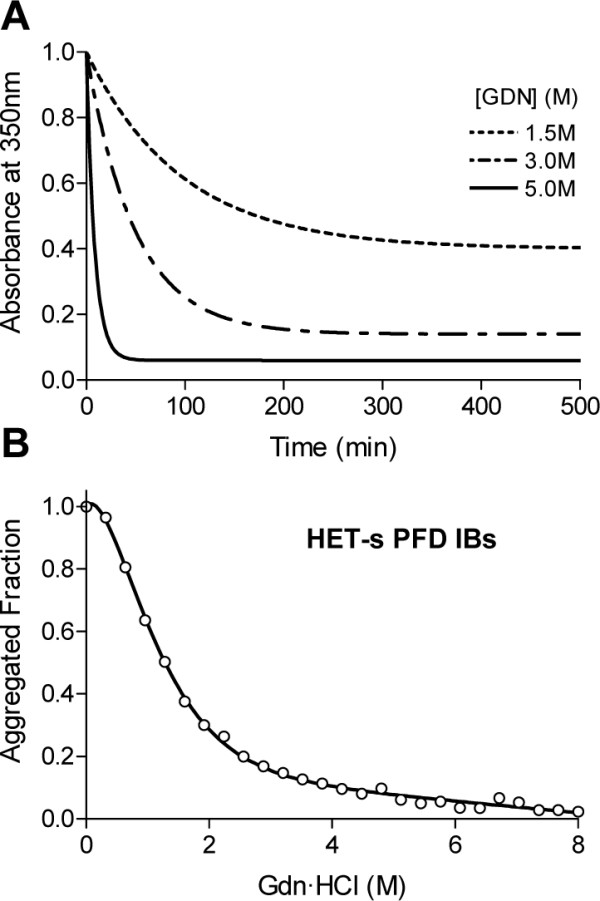
**HET-s IBs stability**. (A) Kinetics of chemical solubilization of HET-s PFD IBs at different Gdn·HCl, monitored by a time dependent decrease in light scattering at 350. (B) Solubilization at the equilibrium of HET-s PFD IBs in the presence of increasing concentrations of Gdn·HCl monitored by light scattering at 350 nm.

We have previously characterized the stability of HET-s PFD fibrils towards chemical denaturation with Gdn·HCl. At pH 7, we found that the midpoint (m_1/2_) of the transition between the aggregated and soluble states of the prion domain is attained at a Gdn·HCl concentration of ~3.5 M [14]. In a recent study we demonstrated that the same approach can be used to approximate the stability of bacterial IBs [23]. This provides us with an opportunity to compare the strength of the contacts stabilizing HET-s PFD IBs with that established by the same polypeptide in the amyloid fibrillar state. IBs denaturation was measured by monitoring the changes in absorbance at 350 nm in the presence of 0 to 8 M Gdn·HCl. As for fibrils, we assumed that only aggregated states contribute significantly to the signal. To determine the incubation time necessary to reach equilibrium we followed the kinetics of IBs solubilization in the presence of different Gdn·HCl concentrations. We observed that, as happens in the denaturation of globular proteins, both the amplitude and the fast rate constant of the reaction increased with increasing chaotropic agent concentrations (Figure 5A). In all cases the reaction was complete before 10 h of incubation. We calculated *m*_1/2 _for IBs solubilization under equilibrium conditions (20 h incubation) to be ~1.5 M (Figure 5B). Therefore, the *in vivo *formed aggregates are significantly less resistant than *in vitro *fibrils to chemical denaturation [14]. The reduced stability of IBs relative to the fibrils is not surprising if we take into account the PK digestion experiments discussed above which show that ordered stable fibrillar and, amorphous and probably less stables structures coexist in IBs. The presence of minor contaminants might also condition the stability of IBs.

### Amyloid seeding capacity of HET-s IBs

**Figure 6:**
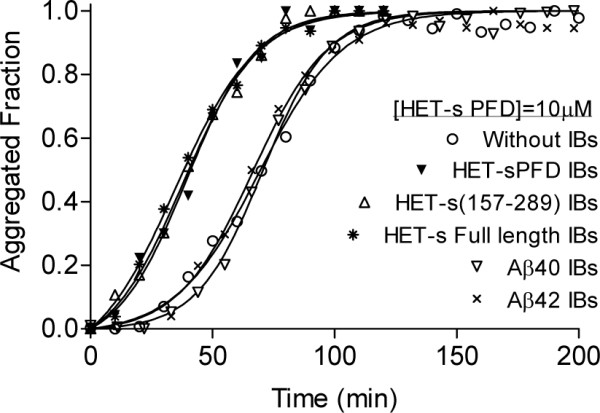
**Seeding-dependent maturation of HET-s PFD amyloid growth**. The aggregation reaction was seeded with HET-s(001-289), HET-s(157-289), HET-s(218-289), Aβ40 or Aβ42 IBs. The fibrillar fraction of HET-s PFD is represented as a function of time. The formation of HET-s PFD amyloid fibrils is accelerated only in the presence of HET-s IBs.

**Figure 7:**
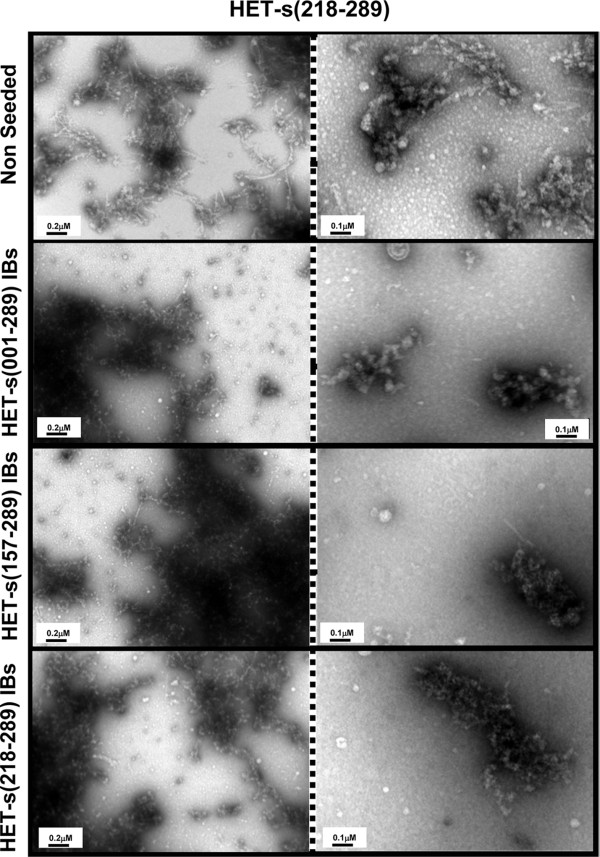
**Morphological properties of HET-s PFD aggregates present at the final time point of soluble monomer non-seeded aggregation reactions (top panel) or at the final stage of reactions seeded with HET-s(001-289) (top-middle panel), HET-s(157-289) (bottom-middle panel) and HET-s(218-289) IBs (bottom panel) as monitored by transmission electronic microscopy**.

The kinetics of amyloid fibril formation usually follow a sigmoidal curve that reflects a nucleation-dependent growth mechanism [24]. The assembly of HET-s PFD fibrils *in vitro *at pH 7.0 follows this kinetic scheme (Figure 6). The detected lag phase corresponds to the formation of the initial nuclei on which the polymerization or fibril growth spontaneously proceeds. This step is considerably shortened by the presence of preformed fibrils than can act as seeds for the polymerization reaction. As previously reported by Wasmer and co-workers [16], the presence of limited amounts of HET-s PFD IBs also promotes a dramatic acceleration in the nucleation rate of soluble HET-s PFD amyloid formation (Figure 6). Interestingly enough, the IBs formed by the full-length prion protein and HET-s 157-298 promote exactly the same effect: the lag phase is shortened from 30 to 10 min and the total reaction time from 110 to 70 min. To ensure that the increase in aggregation rates results from a faster growth of amyloid material and not from the formation of amorphous assemblies, the morphology of the aggregates present in seeded solutions was analyzed by EM at the end of the reaction. As it is shown in Figure 7, independently of the HET-s IBs used to seed the reaction, the presence of abundant fibrillar structures with a morphology closely resembling that of unseeded fibrils was observed in all cases.

Amyloid formation is a highly specific process that can be accelerated only by homologous fibrils, but not by fibrils from unrelated polypeptides. This is because the amino acid sequence dictates the fibril conformation and it is in fact the fibril structure which determines seeding ability [1]. To test if this selectivity also applies in the case of IBs we performed cross-seeding experiments of soluble HET-s PFD with the IBs formed by the two amyloidogenic variants of the Alzheimer's related peptides Aβ40 and Aβ42. The presence of Aβ IBs does not affect the nucleation or the elongation rates (Figure 6) confirming that a highly specific molecular recognition between soluble and aggregated states is indispensable for seeding to occur. Therefore, the cross-reactivity observed for the different HET-s forms strongly suggests that the β-solenoid supramolecular disposition proposed for HET-s PFD IBs [16] is also present in the bacterial aggregates formed by the full-length HET-s prion. Structural similarity of the 218-289 region in PFD alone and full length HET-s *in vitro *amyloids was recently evidenced by ssNMR [25].

## Conclusion

Prions are misfolded, self-propagating, infectious proteins. The HET-s PFD of *Podospora anserina *constitutes the only model for which the infectious fold is known to date at atomic resolution. Moreover, the same fold appears to be conserved in the IBs it forms in bacteria. We show here that the IBs formed by the full-length prion display very similar amyloid characteristics, becoming thus an interesting model to study amyloid formation in bacteria. The formation of prionic infectious folds is known to be tightly controlled by the cellular folding machinery [26]. For HET-s, this propagation depends on molecular chaperones and more specifically on Hsp104 as has been shown not only in *P. anserina *but also when this prion is heterologously expressed in yeast. The data in the present study supports the use bacterial systems to study how the very well characterized homologous prokaryotic chaperones, for example ClpB (a Hsp104 homolog), recombinant eukaryotic chaperones or small chemical compounds modulate the formation and structure of infectious prions in a more simple cellular background. Although solid state NMR provides a detailed view of the conformational properties of IBs at the molecular level, it requires specific equipment, labelling of the proteins, growing of the bacterial cells in non physiological minimal media and is overall a slow technique not suitable for large scale screening. The set of biophysical approaches described in the present work accurately report on the conformational properties of prionic HET-s IBs allowing to monitor how bacterial backgrounds modulate their properties in a faster and simpler manner.

## Methods

### Protein expression

Plasmids encoding for C-terminally histidine-tagged HET-s full-length, HET-s(157-289) and HET-s PFD polypeptides have been previously described [9,11,19]. They were cloned into the NdeI and HindIII sites of the pET21a vector (Novagen) and transformed into BL21(DE3) pLysS cells. The C-terminal histidine tag does not affect the biological activity of HET-s in *P. anserina *[19]. For protein expression, 10 mL overnight culture of transformed cells was used to inoculate 2 L of DYT medium, which was further incubated at 37°C and 200 rpm. At an OD_600 _of 0.5, protein expression was induced with 1 mM of isopropyl-1-thio-β-D-galactopyranoside for 3 h at 37°C, then the cultures were centrifuged and the cell pellet frozen at -20°C.

### IB purification

IBs were purified from induced cell extracts by detergent-based procedures as previously described [27]. Briefly, cells were harvested by centrifugation at 12 000 × *g *(at 4°C) for 15 min and resuspended in 200 μL of lysis buffer (50 mM Tris-Cl pH 8, 1 mM EDTA, 100 mM NaCl), plus 30 μL of 100 mM protease inhibitor PMSF and 6 μL of 10 mg/mL lysozyme. After 30 min of incubation at 37°C under gentle agitation, NP-40 was added at 1% (v/v) and the mixture incubated at 4°C for 30 min. Then, 3 μL of DNase I and RNase from 1 mg/mL stock (25 μg/mL final concentration) and 3 μL of 1 M MgSO_4 _were added and the resulting mixture was further incubated at 37°C for 30 min. Protein aggregates were separated by centrifugation at 12 000 × *g *for 15 min at 4°C. Finally, IBs were washed once with the same buffer containing 0.5% Triton X-100 and once with sterile PBS. After a final centrifugation at 12 000 × *g *for 15 min, pellets were stored at -20°C until analysis. The frozen pellets were reconstituted in PBS buffer. SDS-PAGE analysis revealed that in all cases HET-s proteins were the major polypeptidic components of the respective aggregates.

### Secondary structure determination

ATR FT-IR spectroscopy analyses of HET-s IBs were performed using a Bruker Tensor 27 FT-IR Spectrometer (Bruker Optics Inc) with a Golden Gate MKII ATR accessory. Each spectrum consists of 20 independent scans, measured at a spectral resolution of 2 cm^-1 ^within the 1800-1500 cm^-1 ^range. All spectral data were acquired and normalized using the OPUS MIR Tensor 27 software. Second derivatives of the spectra were used to determine the frequencies at which the different spectral components were located.

CD spectra were collected in the 200 - 250 nm range at 25°C and measured at a spectral resolution of 1 cm^-1^, and a scan rate of 15 nm min^-1 ^using a Jasco 810 spectropolarimeter with a quartz cell of 0.1 cm path length.

### Chemical denaturation

For stability assays, purified IBs were prepared at OD_350 nm _= 1 in PBS solution at pH 7.0 containing selected concentrations of guanidine hydrochloride (Gdn·HCl) ranging from 0 to 8 M. For equilibrium denaturation experiments, the reactions were allowed to reach equilibrium by incubating them for 20 h at room temperature. The fraction of soluble protein (f_S_) was calculated from the fitted values using equation: f_S _= 1-((*y*_S_-*y*)/(*y*_S_-*y*_A_)), where *y*_S _and *y*_A _are the absorbance at 350 nm of the soluble and aggregated protein, respectively, and *y *is the absorbance of the protein solution as a function of denaturant concentration. The value m_1/2 _was calculated as the denaturant concentration at which f_S _= 1/2. OD_350 nm _changes were monitored in a Cary-400 Varian spectrophotometer (Varian Inc.).

For kinetic experiments, purified IBs were prepared at OD_350 nm _= 1 in PBS solution at pH 7.0 containing selected concentrations of Gdn·HCl. The reaction was monitored by measuring the changes in OD_350 nm_. Double-exponential decay curves were fitted to the data using Sigmaplot non-linear regression software (Jandel Scientific, San Rafael, CA, USA), and apparent rate constants were derived from these regressions.

### Limited proteolysis

HET-s IBs (to a final OD_350 nm _= 0.125) were digested at 37°C with 10 μg/mL of proteinase K (PK) in pH 7.0 PBS buffer and the digestion was followed by UV/Vis spectroscopy at 350 nm. After 30 min of reaction, fractions of the samples were centrifuged and the insoluble part resuspended in water, placed on carbon-coated copper grids, and left to stand for five minutes. The grids were washed with distilled water and stained with 2% (w/v) uranyl acetate for another two minutes before analysis using a HitachiH-7000 transmission electron microscope operating at accelerating voltage of 75 kV.

### Congo Red binding

Congo-Red (CR) interaction with different HET-s IBs was tested using a Cary100 (Varian) UV/Vis spectrophotometer by recording the absorbance spectra from 375 nm to 675 nm using a matched pair of quartz cuvettes of 1 cm optical length placed in a thermostated cell holder at 25°C. In order to detect the typical amyloid band at ~540 nm, differential CR spectra in the presence and absence of protein were used.

HET-s IBs were incubated for 1 h in the presence of 50 μM CR. After centrifugation (14 000 × *g *for 5 min), the precipitated fraction was placed on a microscope slide and sealed. The CR birefringence was detected under cross-polarized light using an optic microscope (Leica DMRB, Heidelberg, Germany).

### Seeding assays

HET-s PFD aggregation from soluble monomer was monitored by measuring the transition from non-aggregated to aggregated state by UV/Vis spectrophotometry at 350 nm. In the seeding assay, a solution of different HET-s IBs (to a final OD_350 _= 0.125) was also added at the beginning of the reaction. Cross-seeding assays with Aβ40 and Aβ42 IBs were performed in the same manner. All experiments were carried out at 40°C and 1400 rpm with an initial soluble monomer concentration of 10 μM.

HET-s PFD aggregation process may be studied as an autocatalytic reaction using the equation *f *= (*ρ*{exp [(1+*ρ*)*k*t]-1})/{1+ρ*exp[(1+*ρ*)*k*t]} under the boundary condition of *t *= 0 and *f *= 0, where *k *= *k*_e_a (when a is the protein concentration) and *ρ *represents the dimensionless value to describe the ratio of *k*_n _to *k*. By non-linear regression of *f *against *t*, values of *ρ *and *k *can be easily obtained, and from them the rate constants, *k*_e _(elongation constant) and *k*_n _(nucleation constant). The extrapolation of the growth portion of the sigmoid curve to abscissa (*f *= 0), and to the highest ordinate value of the fitted plot, afforded two values of time (*t*_0 _and *t*_1_), which correspond to the lag time and to the time at which the aggregation was almost complete [28].

## Competing interests

The authors declare that they have no competing interests.

## Authors' contributions

RS and SV directed the work and prepared the manuscript, AE performed experimental work and SJS contributed to the analysis and interpreation of the data.

## Supplementary Material

Additional file 1 **Effect of Proteinase K on HET-s PFD soluble monomer aggregation**. Aggregation of HET-s PFD soluble monomer in the absence (top panel) and presence of 5 μg/mL of proteinase K (bottom panel) as imaged by electronic microscopy. The aggregation assay was realized at 37°C and pH7 for 24 h. Click here for file
