# Supplementary figures and images for: Characterization of the amyloid bacterial inclusion bodies of the HET-s fungal prion
Source: Microb Cell Fact. 2009 Oct 28;8:56. doi: 10.1186/1475-2859-8-56 (PMC2774669; doi:10.1186/1475-2859-8-56)

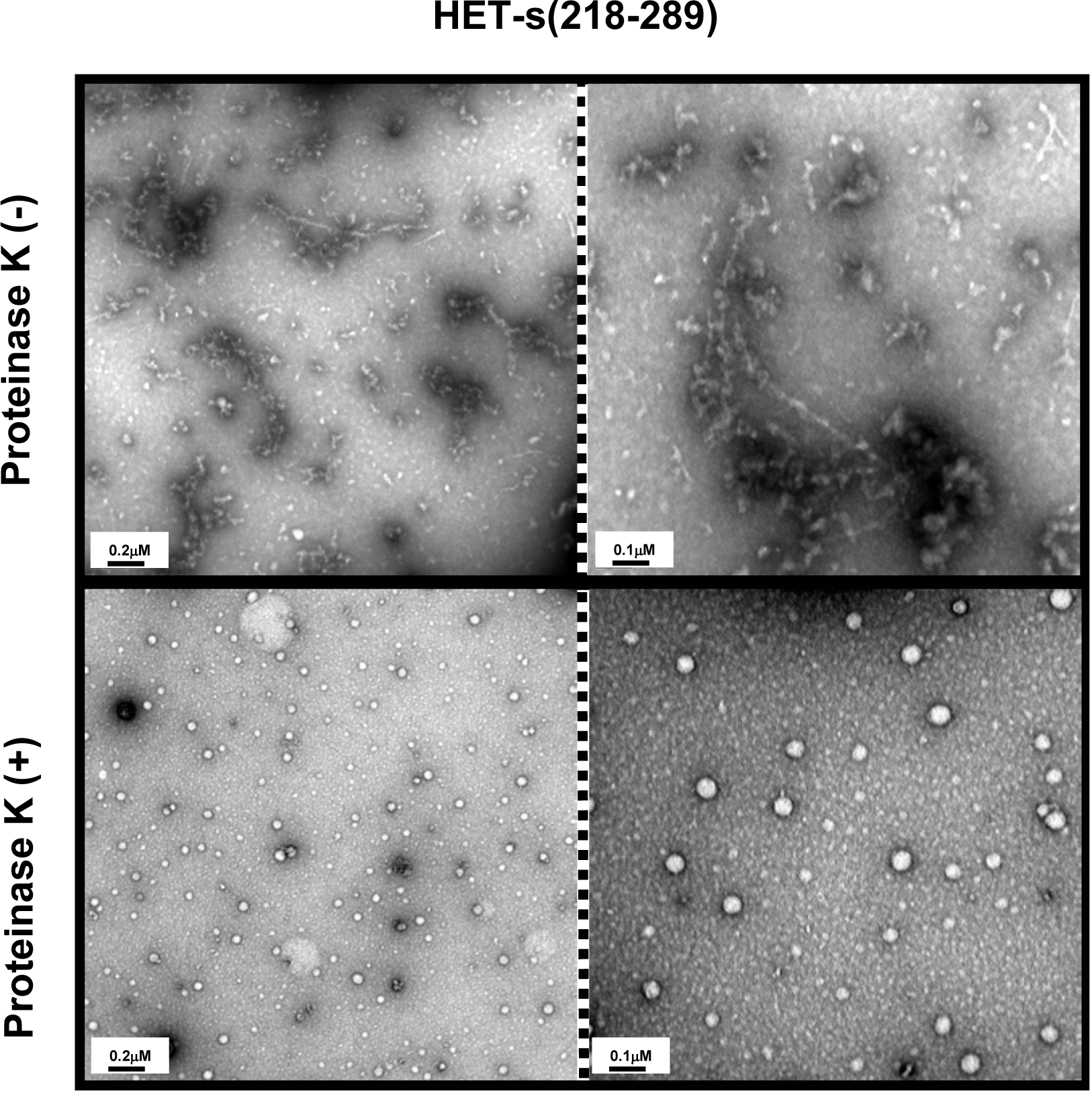

Supplement: Additional file 1 — Effect of Proteinase K on HET-s PFD soluble monomer aggregation. Aggregation of HET-s PFD soluble monomer in the absence (top panel) and presence of 5 μg/mL of proteinase K (bottom panel) as imaged by electronic microscopy. The aggregation assay was realized at 37°C and pH7 for 24 h. [file 1475-2859-8-56-S1.TIFF]
